# A Mannose Receptor from *Litopenaeus vannamei* Involved in Innate Immunity by Pathogen Recognition and Inflammation Regulation

**DOI:** 10.3390/ijms241310665

**Published:** 2023-06-26

**Authors:** Na Guo, Yuan Liu, Qiang Hao, Mingzhe Sun, Fuhua Li

**Affiliations:** 1 School of Marine Science and Engineering, Qingdao Agricultural University, Qingdao 266109, China; guona1291417537@163.com (N.G.); qiangh1029@163.com (Q.H.); 2 Shandong Province Key Laboratory of Experimental Marine Biology, Institute of Oceanology, Chinese Academy of Sciences, Qingdao 266071, China; mzhsun@qdio.ac.cn (M.S.); fhli@qdio.ac.cn (F.L.); 3 Center for Ocean Mega-Science, Chinese Academy of Sciences, Qingdao 266071, China; 4 The Innovation of Seed Design, Chinese Academy of Sciences, Wuhan 430072, China

**Keywords:** mannose receptor, *Litopenaeus vannamei*, pathogen recognition, inflammation, ROS

## Abstract

Mannose receptor, as a member of the C-type lectin superfamily, is a non-canonical pattern recognition receptor that can internalize pathogen-associated ligands and activate intracellular signaling. Here, a mannose receptor gene, LvMR, was identified from the Pacific white shrimp *Litopenaeus vannamei*. LvMR encoded a signal peptide, a fibronectin type II (FN II) domain, and two carbohydrate-recognition domains (CRDs) with special EPS and FND motifs. LvMR transcripts were mainly detected in the hepatopancreas, and presented a time-dependent response after pathogen challenge. The recombinant LvMR (rLvMR) could bind to various PAMPs and agglutinate microorganisms in a Ca^2+^-dependent manner with strong binding ability to _D_-mannose and *N*-acetyl sugars. The knockdown of LvMR enhanced the expression of most NF-κB pathway genes, inflammation and redox genes, while it had no obvious effect on the transcription of most phagocytosis genes. Moreover, the knockdown of LvMR caused an increase in reactive oxygen species (ROS) content and inducible nitric oxide synthase (iNOS) activity in the hepatopancreas after *Vibrio parahaemolyticus* infection. All these results indicate that LvMR might perform as a PRR in immune recognition and a negative regulator of inflammation during bacterial infection.

## 1. Introduction

Mannose receptor (MR) is a unique multidomain and multifunctional pattern recognition receptor (PRR) belonging to the C-type lectin superfamily [1]. In mammals, MR, primarily expressed by macrophages and dendritic cells, is a typical type I transmembrane receptor consisting of a cysteine-rich (CR) domain, a single fibronectin type II (FN II) domain, eight C-type carbohydrate-recognition domains (CRDs), a transmembrane region and a short cytoplasmic region [2,3]. It can recognize and internalize endogenous and pathogen-associated ligands, and play important roles in antigen processing and presentation, inflammation and intracellular signal transduction [4].

MR was initially identified in rabbit alveolar macrophages as a 175 kDa transmembrane receptor involved in the clearance of endogenous glycoproteins [5]. Thereafter, the structure and function of MRs have been described extensively in mammals. Due to the crucial role in both innate and acquired immune responses, many studies about MRs has been recently conducted in some aquatic animals, such as the zebra fish (*Danio rerio*) [6], large yellow croaker (*Larimichthys crocea*) [7], blunt snout bream (*Megalobrama amblycephala*) [8] and Nile tilapia (*Oreochromis niloticus*) [9]. Expression analysis showed that the transcripts of fish MRs could be significantly induced in response to pathogenic infection [6,9]. Functional analysis revealed that the OnMR of Nile tilapia and the MaMR of Asian swamp eel could bind and agglutinate Gram-positive and Gram-negative bacteria [9,10]. Blunt snout bream MR was reported to be involved in the phagocytosis of macrophage to bacteria, accompanied by respiratory burst, nitric oxide (NO) production and inflammatory cytokines secretion [8]. To date, invertebrate MR has only been identified in the red swamp crayfish *Procambarus clarkii*, which contained 14 CTLDs and a transmembrane domain [11]. Knowledge about the structure and function of MRs in invertebrates is very limited.

The Pacific white shrimp *Litopenaeus vannamei* is one of the most important commercial aquaculture species in the world. However, infections with bacteria and viruses cause great losses to the shrimp aquaculture industry [12]. To further investigate the immune defense in shrimp, here, a novel MR gene from *L. vannamei*, designed as LvMR, was identified and characterized. The expression patterns of the LvMR of tissue distribution and the after-pathogen challenge were detected. The PAMP recognition and microorganism agglutination of the recombinant LvMR (rLvMR) were studied. In the case of a LvMR knockdown by double-stranded RNA (dsRNA), the roles of the LvMR in regulating phagocytosis, inflammation and immune signaling transduction were investigated. Specifically, the involvement of LvMR in regulating oxidative stress in the hepatopancreas induced by *Vibrio parahaemolyticus* was determined by measuring redox genes expression, ROS content and iNOS activity.

## 2. Results

### 2.1. cDNA Cloning and Sequence Analysis of LvMR

**Figure 1:**
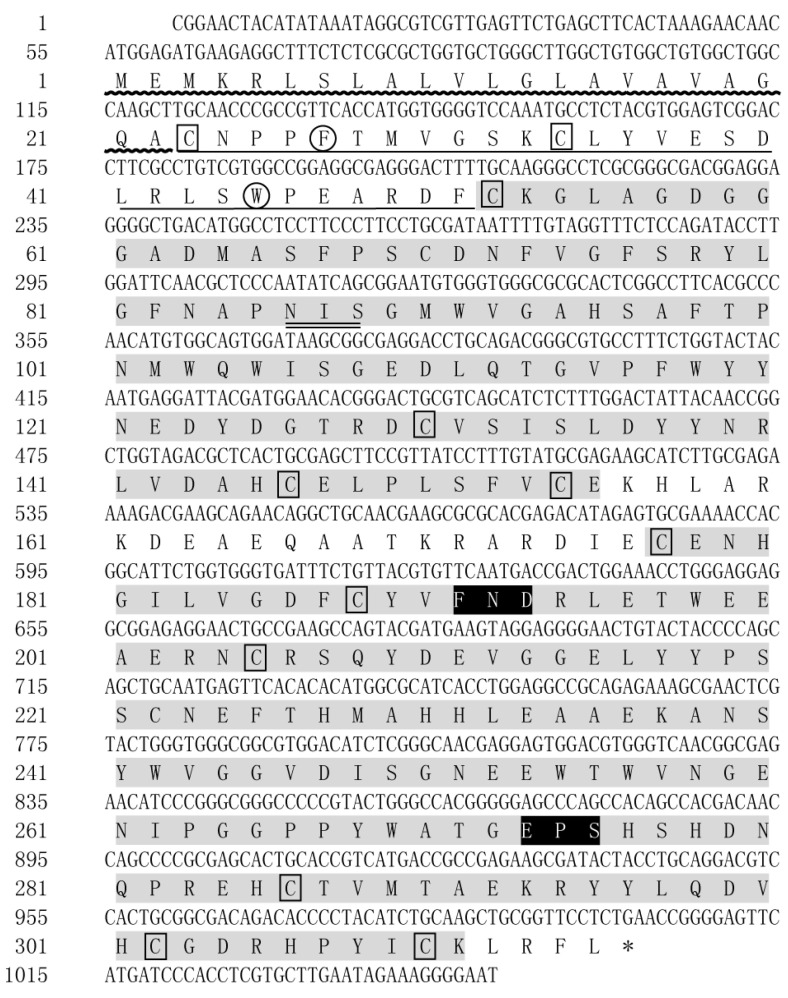
Nucleotide and deduced amino acid sequences of LvMR from *L. vannamei*. The stop codon (TGA) is marked with asterisk (*). The signal peptide is indicated with wavy line. The FN II domain is underlined, and the CRDs are shaded with gray. Two conserved aromatic residues are circled, and conserved cysteine residues are boxed. The motifs for carbohydrate-binding are shaded with black. The glycosylation sites are double-underlined.

The cDNA sequence of LvMR was cloned from the hepatopancreas, and submitted to GenBank under the accession no. OR051718. The obtained sequence was 1049 bp in length, and contained a 54 bp 5′-untranslated region (UTR), a 948 bp open reading frame (ORF), and a 47 bp 3′-UTR (Figure 1). LvMR was predicted to encode a polypeptide of 315 amino acid residues, including a signal peptide, a fibronectin type II (FN II) domain and two C-type carbohydrate-recognition domains (CRDs). The FN II domain consisted of two conserved cysteines (Cys^23^, Cys^34^) and two conserved aromatic residues (Phe^27^, Trp^46^). The two CRD domains shared 23.0% identity with each other. The first CRD had four conserved cysteine residues (Cys^52^, Cys^130^, Cys^146^, Cys^154^) and one potential *N*-linked glycosylation modification site (Asn^86^-Ile^87^-Ser^88^). The second CRD contained four conserved cysteine residues (Cys^205^, Cys^286^, Cys^302^, Cys^310^), forming two internal disulfide bridges, and two additional cysteine residues (Cys^177^, Cys^188^) at the N-terminal end. Two potential carbohydrate-binding motifs FND (Phe^191^-Asn^192^-Asp^193^) and EPS (Glu^273^-Pro^274^-Ser^275^) were detected in the second CRD. The mature LvMR was estimated to be 33.48 kDa with a theoretical isoelectric point of 4.77.

### 2.2. Homology and Phylogenetic Analysis of LvMR

**Figure 2:**
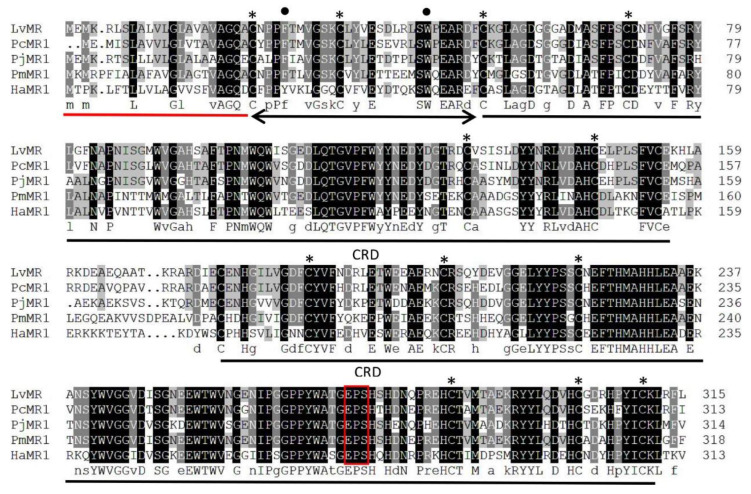
Multiple alignment of LvMR with other crustacean MRs. Identical and similar residues were shaded in black and gray, respectively. The signal peptide is underlined in red, and the FN II domain is indicated by black line with arrows. Two CRD domains are underlined in black. Conserved aromatic residues and cysteine residues are marked with dots and asterisks. The potential carbohydrate-binding motif is boxed in red. The used sequences and the GenBank accession numbers are as follows: PcMR1 (*P. chinensis*, XP_047470230.1), PjMR1 (*P. japonicus*, XP_042890295.1), PmMR1 (*P. monodon*, XP_037780720.1), HaMR1 (*H. americanus*, XP_042237421.1).

**Figure 3:**
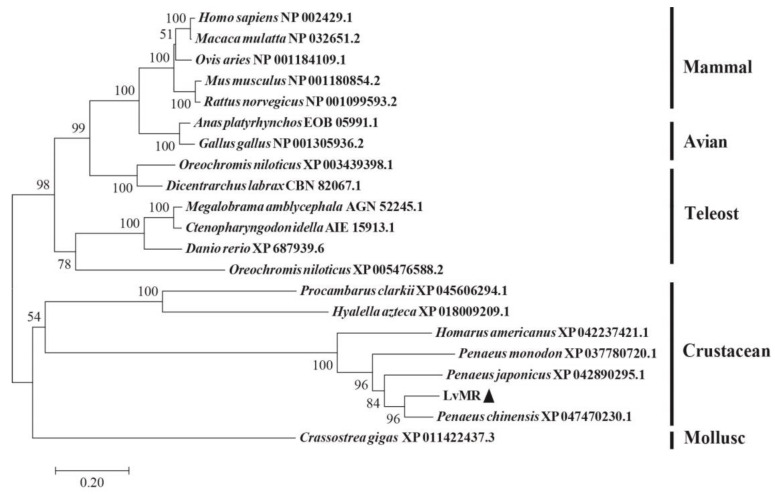
Neighbor-joining phylogenetic tree of representative MRs. Numbers at each branch indicated bootstrap values of 1000 replicates. The bar (0.2) indicated the genetic distance. LvMR was marked by ▲.

Homology analysis indicated that the deduced amino acid sequence of LvMR shared high similarity with other crustacean MRs (Figure 2). LvMR showed 82.2% identity with PcMR1 (XP_047470230.1) from *Penaeus chinensis*, 74.2% identity with PjMR1 (XP_042890295.1) from *Penaeus japonicus*, 64.4% identity with PmMR1 (XP_037780720.1) from *Penaeus monodon*, and 54.6% identity with HaMR1 (XP_042237421.1) from *Homarus americanus*. Except HaMR1, all these MRs had a FN II domain, with two conserved aromatic residues and two CRDs containing conserved cysteine residues and EPS motif. The phylogenetic tree constructed, based on 21 amino acid sequences of MR, could be divided into two clades. MRs from shrimp (*L. vannamei*, *P. chinensis*, *P. japonicus* and *P. monodon*), lobster (*H. americanus*), crayfish (*P. clarkii*), amphipoda (*Hyalella azteca*) and oyster (*Crassostrea gigas*) formed the invertebrate clade, and those from mammals, birds and fish formed the vertebrate clade. LvMR was clustered in a crustacean clade, and had a closer relationship with MRs of *P. chinensis*, *P. japonicus* and *P. monodon* (Figure 3).

### 2.3. Tissue Expression and Immune Responses of LvMR

**Figure 4:**
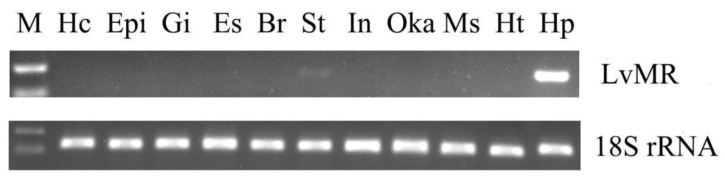
Tissue distribution of LvMR. The 18S rRNA gene was used as the internal reference. Hc, hemocytes; Epi, epidermis; Gi, gill; Es, eyestalk; Br, brain; St, stomach; In, intestine; Oka, lymphoid organ; Ms, muscle; Ht, heart; Hp, hepatopancreas.

**Figure 5:**
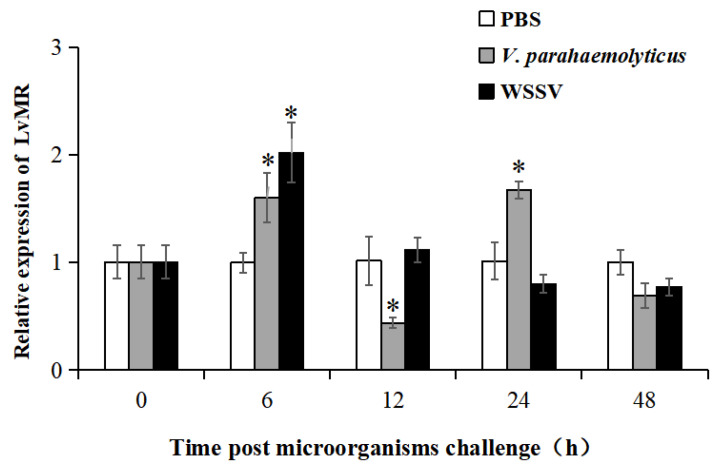
Relative expression of LvMR after challenge with *V. parahaemolyticus* (gray bars) and WSSV (black bars). Data are represented as mean ± S.D. (*n* = 3). Asterisks indicate the significant differences between the experiment group and the control group at the same sampling point (* *p* < 0.05).

Tissue distribution analysis showed that LvMR transcripts were detected mostly in the hepatopancreas, with a few in the stomach, but not in other examined tissues (Figure 4). The temporal expression of LvMR in the hepatopancreas was investigated post *V. parahaemolyticus* and WSSV stimulation (Figure 5). After *V. parahaemolyticus* infection, the expression of LvMR could be rapidly upregulated at 6 h post-injection (1.60-fold to the control group, *p* < 0.05), and decreased significantly at 12 h post-injection (0.43-fold to the control group, *p* < 0.05). As the time progressed, the expression level was upregulated again, and peaked at 24 h post *V. parahaemolyticus* challenge (1.67-fold relative to the control group, *p* < 0.05). After WSSV injection, the expression of LvMR was upregulated and peaked at 6 h post-injection, which was 2.01-fold relative to the control group (*p* < 0.05). Afterward, LvMR expression dropped and recovered to the control level during the following 48 h post-injection.

### 2.4. Expression and Purification of the Recombinant LvMR

**Figure 6:**
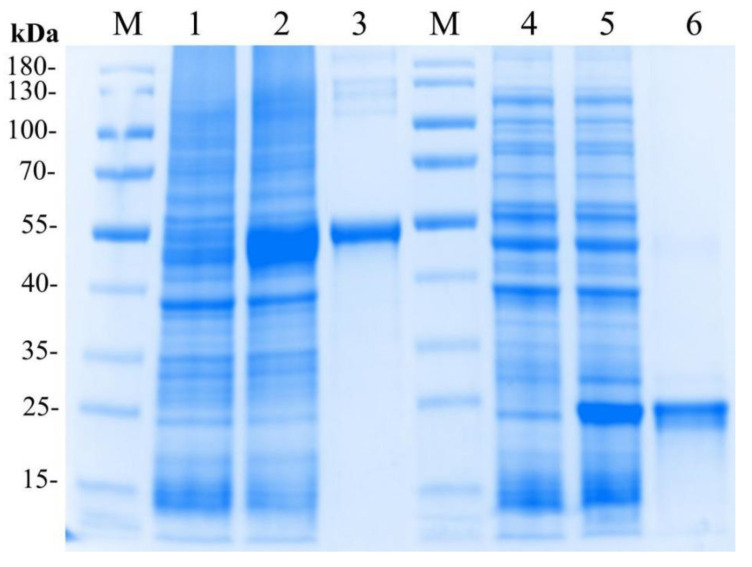
SDS-PAGE analysis of rLvMR and rTrx. M, protein molecular standard (kDa); lane 1, total protein of *E. coli* without recombinant plasmid transformed of pET32a-LvMR; lane 2, total protein of *E. coli* with recombinant plasmid of pET32a-LvMR after IPTG induction; lane 3, purified recombinant protein LvMR; lane 4, total protein of *E. coli* without recombinant plasmid of pET32; lane 5, total protein of *E. coli* with recombinant plasmid of pET32a after IPTG induction; lane 6, purified recombinant protein pET32a.

The recombinant plasmid pET32a-LvMR was transformed and expressed in *E. coli* BL21 (DE3). After IPTG induction, the whole cell lysate was analyzed by SDS-PAGE (Figure 6). The recombinant protein LvMR, expressed in the inclusion body, had a distinct band with molecular mass of about 53 kDa, which was consistent with the predicted molecular mass of fusion protein. Meanwhile, a control rTrx was successfully expressed and found to be 20 kDa. The concentration of the rLvMR and rTrx proteins was 1.5 mg mL^−1^ and 1.0 mg mL^−1^, respectively.

### 2.5. Binding Activity of rLvMR to PAMPs

**Figure 7:**
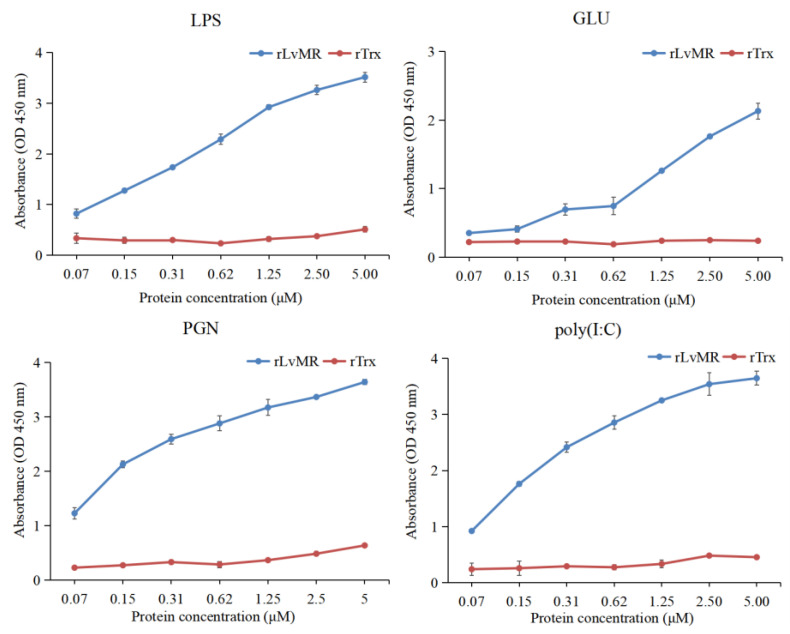
ELISA analysis of the interaction between rLvMR and PAMPs (LPS for lipopolysaccharide, GLU for glucan, PGN for peptidoglycan, poly(I:C) for polyinosinic: polycytidylic acid). The pET32a empty vector expressed protein (rTrx) was used as the negative control. Data are shown as mean ± S.D. (*n* = 3).

The binding activity of rLvMR towards various PAMPs was detected by ELISA (Figure 7). The results showed that rLvMR could bind GLU, LPS, PGN and poly(I:C), and the binding ability increased gradually with the increase in rLvMR concentration. As a control protein, rTrx could not bind to any tested PAMPs.

### 2.6. Agglutinating Activity of rLvMR to Microorganisms

**Figure 8:**
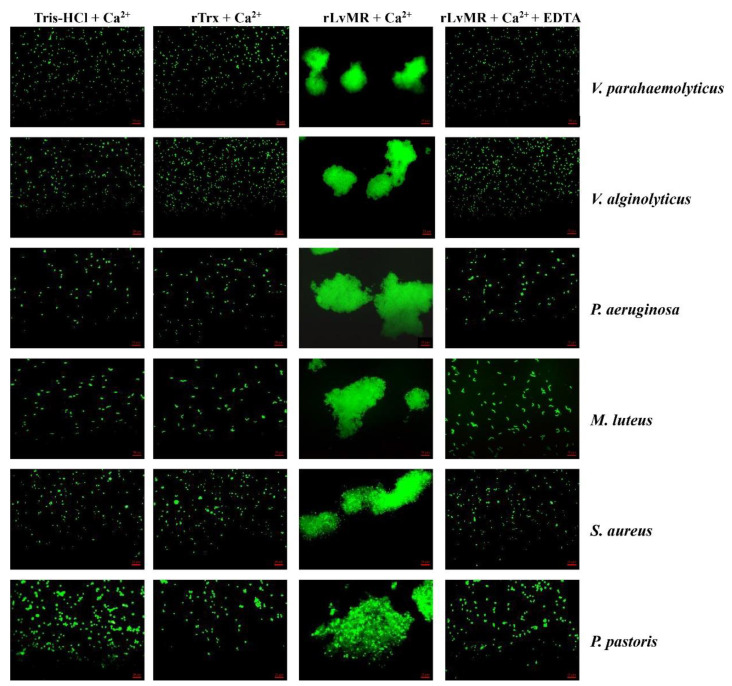
Agglutination of FITC-labeled bacteria and fungus induced by rLvMR. The FITC-labeled microorganisms (*Vibrio alginolyticus*, *V. parahaemolyticus*, *Pseudomonas aeruginosa*, *Micrococcus luteus*, *Staphylococcus aureus* and *Pichia pastoris*) were incubated with rLvMR or rTrx in the presence of 10 mM CaCl_2_ or 10 mM EDTA. Tris-HCl and rTrx were used as blank and negative control, respectively.

**Figure 9:**
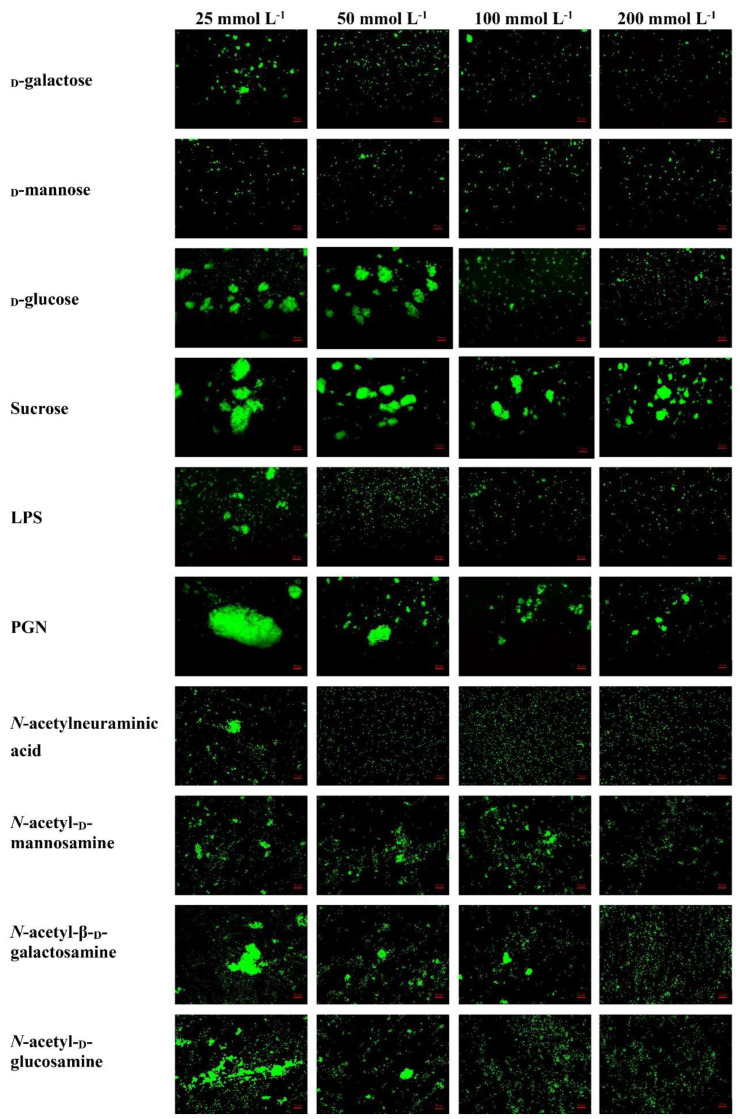
Inhibition of agglutinating activity of rLvMR towards FITC−labeled *V. parahaemolyticus* by different carbohydrates. LvMR and different concentrations of carbohydrates were incubated with FITC−labeled *V. parahaemolyticus* in the presence of 10 mM CaCl_2_.

The rLvMR could agglutinate the tested FITC-labeled bacteria and fungus in the presence of Ca^2+^ (Figure 8). The agglutination effect disappeared when EDTA was added. There was no agglutination observed in the negative control (rTrx) and the blank control (Tris-HCl) in the presence of Ca^2+^. The agglutination of rLvMR towards *V. parahaemolyticus* was inhibited with incubation of 25 mmol L^−1^ _D_-mannose, 50 mmol L^−1^ _D_-galactose, 50 mmol L^−1^ LPS, 50 mmol L^−1^ *N*-acetylneuraminic acid, 100 mmol L^−1^ _D_-glucose, 100 mmol L^−1^ *N*-acetyl-_D_-glucosamine, 100 mmol L^−1^ PGN, 200 mmol L^−1^ *N*-acetyl-_D_-mannosamine and 200 mmol L^−1^ *N*-acetyl-β-_D_-galactosamine (Figure 9). Additionally, the inhibition of agglutinating activity was enhanced by the increasing concentration of LPS, _D_-galactose, *N*-acetylneuraminic acid, _D_-glucose, *N*-acetyl-_D_-glucosamine, *N*-acetyl-β-_D_-galactosamine and PGN. However, the agglutination effect did not change obviously with the addition of sucrose at its maximum tested concentrations.

### 2.7. Gene Knockdown of LvMR

**Figure 10:**
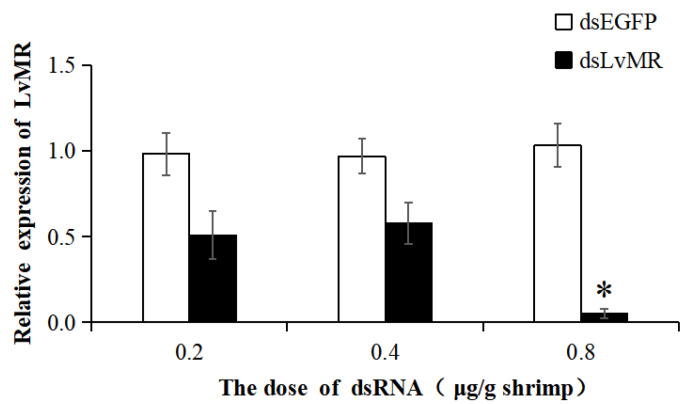
The knockdown efficiency of LvMR in shrimp hepatopancrease by 48 h post dsRNA injection. Data are represented as mean ± S.D. (*n* = 3). Significant differences across control is indicated with one asterisk (*p* < 0.05).

The dsRNA-induced RNAi was used to knock down the expression of LvMR. Compared with the EGFP-dsRNA group, the expression of LvMR was significantly downregulated in the hepatopancrease of shrimp injected with dsLvMR at 0.8 μg dsRNA/g shrimp, and the knockdown efficiency of LvMR was 94.8% (Figure 10). However, no significant inhibitory effect was found on shrimp injected with dsLvMR at 0.2 μg dsRNA/g shrimp and 0.4 μg dsRNA/g shrimp. Therefore, 0.8 μg dsLvMR/g shrimp was selected as the optimal dose for further interference experiments.

### 2.8. Effects of LvMR Interference on the Expression of Immune Genes

**Figure 11:**
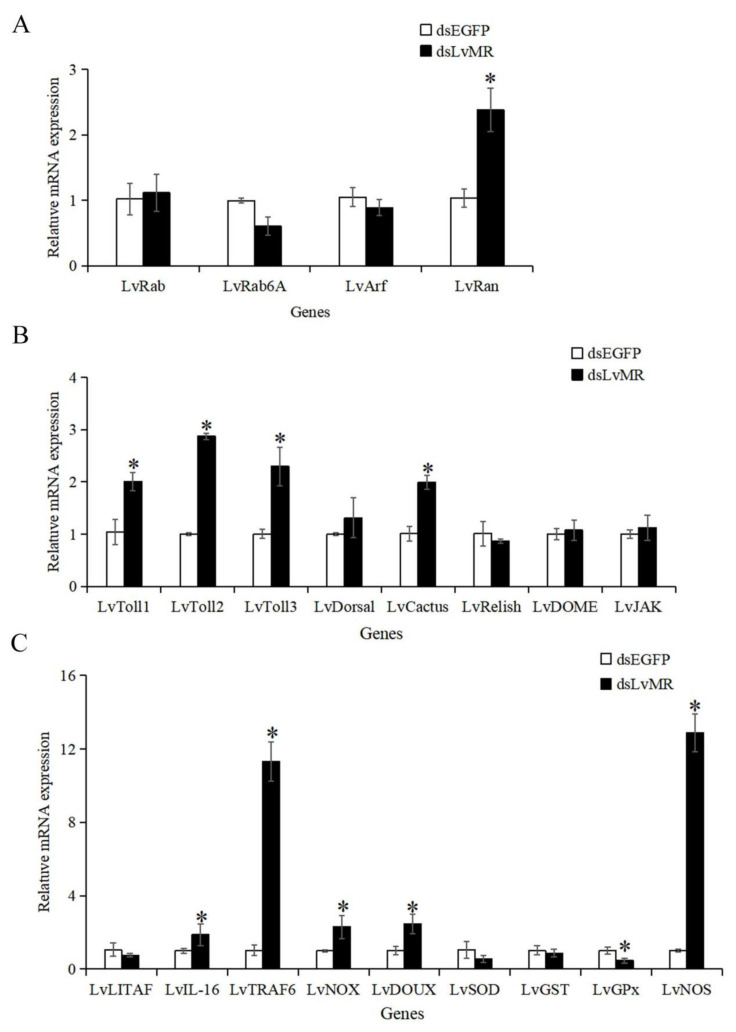
Expression pattern of immune genes after LvMR RNAi. The immune genes are involved in (**A**) phagocytosis, (**B**) NF-κB and JAK/STAT signaling pathways, and (**C**) inflammation and redox. Data are shown as mean ± S.D. (*n* = 3). Asterisks indicate the significant differences compared with control at the same sampling point (*p* < 0.05).

After the LvMR gene knockdown, only the transcription of the redox gene LvGPx was significantly downregulated. The expression levels of the phagocytosis gene LvRan, NF-κB pathway genes LvToll1-3 and LvCactus, inflammation-related genes LvIL-16 and LvTRAF6, and redox genes LvNOX, LvDOUX and LvNOS in the dsLvMR group were significantly upregulated, compared with the dsEGFP group (Figure 11). There was no obvious effect on the expression of the LvLITAF, LvSOD, LvGST, other phagocytosis genes LvRab, LvRab6A and LvArf, and JAK/STAT pathway genes LvJAK and LvDOME after the knockdown of LvMR.

### 2.9. Effects of LvMR Interference on ROS Production

**Figure 12:**
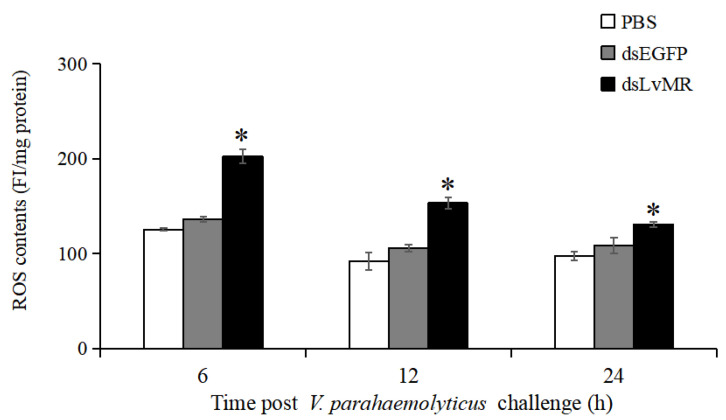
ROS production in the hepatopancreas cells of dsRNA injected shrimp in response to *V. parahaemolyticus* infection at different time points. Data are shown as mean ± S.D. (*n* = 3). Significant differences across control is indicated with one asterisk (*p* < 0.05).

After LvMR knockdown, ROS production in the hepatopancreas increased significantly in the *V. parahaemolyticus*-infected shrimp compared with the control (*p* < 0.05, Figure 12). The highest ROS level was observed in the LvMR-knockdown shrimp at 6 h post *V. parahaemolyticus* injection, which was 32.6% greater than that of the dsEGFP group. As time progressed, the *V. parahaemolyticus*-induced ROS levels decreased gradually in the LvMR-knockdown shrimp.

### 2.10. Effects of LvMR Interference on iNOS Activity

**Figure 13:**
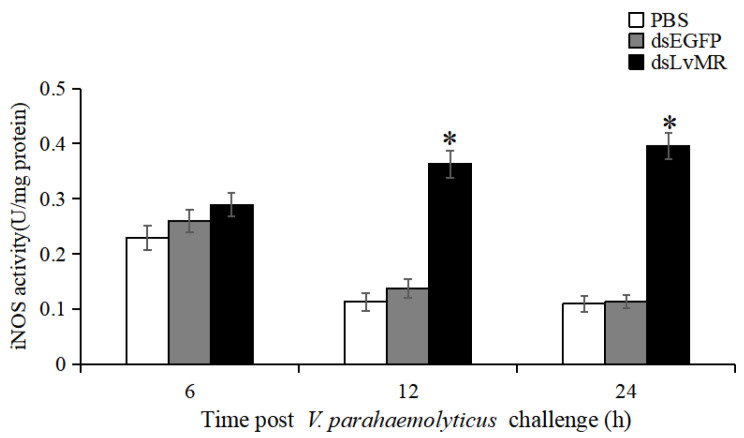
iNOS activity in the hepatopancreas cells of dsRNA injected shrimp in response to *V. parahaemolyticus* infection at different time points. Data are shown as mean ± S.D. (*n* = 3). Significant differences across control is indicated with one asterisk (*p* < 0.05).

The involvement of LvMR in regulating NO production was verified by measuring iNOS activity in the *V. parahaemolyticus*-induced shrimp. Compared to the dsEGFP group, iNOS activity in the hepatopancrease of LvMR-knockdown shrimp were significantly upregulated at 12 and 24 h post *V. parahaemolyticus* injection, which was increased by 62.1% and 71.4%, respectively (*p* < 0.05, Figure 13). No significant differences were found between the LvMR-knockdown group and control groups at 6 h post *V. parahaemolyticus* injection.

## 3. Discussion

In the present study, a mannose receptor named LvMR was identified and characterized from *L. vannamei*. The vertebrate MRs share a common structure, including extracellular CR domain, FN II domain and eight tandemly arranged CRDs, transmembrane and cytoplasmic regions [13]. Different from the vertebrate MRs, LvMR had only the FN II domain and two CRDs. This is similar to the reported PcMR in *P. clarkii* [11] and other invertebrate MRs available in GenBank, such as MRs from *H. azteca*, *C. gigas* and *Lingula anatina*, which had 12–15 CRDs with the absence of CR and FNII domains. The structural differences indicate that the domains of MRs are not conserved in invertebrates. The FN II domain, important for collagen binding, is the most conserved domain among members of the MR family [3,14]. Multiple sequence alignment analysis showed that the FN II domains were conserved in some crustacean MRs, suggesting the evolutionary conservation of the MR family. Both homology and phylogenetic analysis showed that LvMR displayed high sequence similarity and close evolutionary relationship with other MRs, suggesting LvMR is a new invertebrate MR.

EPN (Glu-Pro-Asn) and WND (Trp-Asn-Asp) are the classical carbohydrate-binding motifs in vertebrate C-type lectin-like domains [15]. However, LvMR possessed the mutated motifs EPS and FND in the second CRD, which is similar to that reported in other invertebrate C-type lectins, such as AiCTL-7 of *Argopectens irradians* with the EPD motif [16], MjLecA of *Marsupenaeus japonicus* with the EPS motif [17], FmLC of *Fenneropenaeus merguiensis* with the MND motif [18] and PtCLec1 of *Portunus trituberculatus* with FND [19]. These diverse ligand-binding motifs might change the specificity of carbohydrate recognition, and might be a strategy for invertebrates to recognize diverse pathogens.

Consistent with that reported in the PcMR of *P. clarkii* [11], LvMR was predominantly expressed in the hepatopancreas of *L. vannamei*. The hepatopancreas is not only a digestive gland, but also an important organ involved in the immune response of crustaceans [20,21]. The highest expression of LvMR in the hepatopancreas may be related to the clearance of pathogens in shrimp. It is further supported by the expression pattern of LvMR after a pathogen challenge, which was significantly up-regulated at 6 h post-injection. Additionally, LvMR was increased significantly until 24 h after the *V. parahaemolyticus* challenge, suggesting that LvMR might provide long-lasting protection against invading *V. parahaemolyticus* compared to WSSV.

The PAMP binding assay was performed to verify the role of LvMR as PRR. The successfully expressed rLvMR could bind to LPS, GLU, PGN and poly(I:C) in a dose-dependent manner, which is similar to those reported in the rPcMR-CTLDs from *P. clarkii* [11] and rMaMR-CTLD4-8 from *Monopterus albus* [10]. This finding suggests that LvMR could act as a PRR to recognize various PAMPs and initiate the downstream immune response in shrimp. The rLvMR protein agglutinated bacteria and the fungus in the presence of Ca^2+^, indicating that LvMR is a member of the Ca^2+^-dependent mannose receptors. The similar microorganism agglutination has been found in other reported MRs. For example, in the presence of Ca^2+^, the rCTLD1-3 and rCTLD11-14 of PcMR could agglutinate most test bacteria and fungi [11], and rOnMR could agglutinate Streptococcus agalactiae and Aeromonas hydrophila [9].

The extracellular CRDs of MRs display Ca^2+^-dependent binding to terminal mannose, fucose and *N*-acetyl-glucosamine that are frequently found on the pathogen surfaces [22,23]. We tested the carbohydrate specificity of LvMR in the inhibition assay, and found rLvMR had a strong affinity for _D_-mannose, *N*-acetyl-_D_-glucosamine and other carbohydrates, such as LPS, _D_-galactose, _D_-glucose, *N*-acetylneuraminic acid and *N*-acetyl-β-_D_-galactosamine, which might due to the presence of the tripeptide motif in the CRD domain. The EPN motif is recognized as the mannose specificity-determining motif in the C-type lectins [24]. LvMR with the EPS motif had a more diverse carbohydrate recognition specificity, further suggesting that the carbohydrate-binding specificity of MRs is not conserved in invertebrates.

In addition to pathogen recognition, MR, as a ‘non-canonical’ PRR, is shown to participate in intracellular signaling leading to target gene expression with the assistance of other receptors [2,25]. Considering the important function of MR in phagocytosis and inflammation, we decided to examine the expression of phagocytosis genes and inflammation-related NF-κB pathway genes and cytokines in the LvMR-knockdown shrimp. Our results revealed that knockdown of LvMR had no obvious effect on the expression of most phagocytosis genes except LvRan, suggesting the possible role of MR in regulation of phagocytosis by Ran GTPase. Contrary to the most phagocytosis genes, the expression of most NF-κB pathway genes and inflammation genes was significantly increased in the LvMR knockdown shrimp. It is consistent with that reported in the EcMR from orange-spotted grouper *Epinephelus coioides* and mannose receptors on human alveolar macrophage, where MRs knockdown lead to the increase of proinflammatory cytokines [26,27]. These results indicate that LvMR might play an important role in the anti-inflammatory process.

During the inflammatory response, activated neutrophils and macrophages undergo a respiratory burst and generate a large amount of ROS and RNS [13]. Therefore, we investigated the expression of antioxidant enzymes in the LvMR-knockdown shrimp, and the ROS production and iNOS activity in the hepatopancreas after *V. parahaemolyticus* injection. The knockdown of LvMR could upregulate the expression of antioxidant enzymes and increase the ROS content and NOS activity, further confirming the anti-inflammatory role of LvMR in shrimp immune system.

In conclusion, a novel mannose receptor LvMR containing a FN II domain and two CRDs was identified from *L. vannamei*. LvMR was predominantly expressed in the hepatopancreas, and clearly responded to *V. parahaemolyticus* and WSSV infection. The recombinant LvMR had significant binding and agglutinating activities to PAMPs or pathogens. LvMR with a mutated EPS motif had a more diverse carbohydrate recognition specificity. LvMR knockdown could upregulate the expression of most inflammation-related genes and antioxidant enzymes, and enhance the respiratory burst of the hepatopancreas induced by *V. parahaemolyticus* infection by the increase in ROS content and iNOS activity. All these results suggest that LvMR may participate in the immune recognition and anti-inflammatory response in the innate immunity of *L. vannamei.*

## 4. Materials and Methods

### 4.1. Shrimp, Immune Stimulation and Sample Collection

The healthy shrimp *L. vannamei* cultured in our lab were used as the experimental animals. The average weight of the shrimp used for tissue expression pattern, pathogen infection and RNA interference experiments were 9.7 ± 0.46 g, 9.5 ± 0.8 g and 5.0 ± 0.6 g, respectively. Before experiments, the shrimp were cultured for one week in aerated recirculated seawater with a temperature of 25 ± 1 °C, to adapt to the environment.

The samples of hemocytes, lymphoid organs, hepatopancrease, gills, hearts, stomachs, brains, epithelia, eyestalks, intestines and muscles were collected from five healthy shrimp for tissue distribution analysis. Hemolymph was extracted from the ventral sinus located at the first abdominal segment of shrimp using a sterile syringe with an equal volume of precooled anticoagulant solution (115 mmol L^−1^ glucose, 27 mmol L^−1^ sodium citrate, 336 mmol L^−1^ NaCl, 9 mmol L^−1^ EDTA•Na_2_•2H_2_O, pH 7.4). The hemolymph was immediately centrifuged at 1000× *g*, 4 °C for 5 min to separate the hemocytes. The hemocytes were collected at the bottom of the tube and frozen in liquid nitrogen. Other tissues were dissected directly from the shrimp and frozen in liquid nitrogen.

For the immune stimulation experiment, healthy shrimp were randomly divided into three groups, including one control group, one *V. parahaemolyticus* stimulation group and one WSSV stimulation group. *V. parahaemolyticus* and WSSV were prepared and diluted in PBS at a final concentration of 5 × 10^4^ cfu/tail and 1 × 10^3^ copies/tail, respectively. The diluted pathogen was injected into the muscle of the third or fourth ventral segment of the shrimp at a dose of 10 μL/tail using a microsyringe. Hepatopancrease were collected at 0, 6, 12, 24 and 48 h after *V. parahaemolyticus* or WSSV challenge. Shrimp injecting with PBS served as the control group. At each time point, each group had three biological replicates with five shrimp in each replicate.

### 4.2. RNA Extraction, cDNA Synthesis and Gene Cloning

Total RNA was extracted using RNAiso Plus Reagent (TaKaRa, Dalian, China) from different tissues, according to the manufacturer’s instruction. The concentration and purity of total RNA were measured by Nanodrop 2000 spectrophotometer (Thermo Fisher Scientific, Waltham, MA, USA) and its quality was checked by 1% agarose gel electrophoresis. The first-strand cDNA was synthesized using the PrimeScript™ II 1st cDNA Synthesis Kit (TaKaRa, Dalian, China).

Based on the unigene sequence obtained from our transcriptome, a pair of primers, LvMR_F and LvMR_R (Table 1), were designed to amplify the open reading frame of LvMR. The polymerase chain reaction (PCR) was conducted under the following parameters: a 25 μL reaction volume containing 9.5 μL sterile distilled H_2_O, 12.5 μL of 2 × Accurate Taq Master Mix (Accurate Biotechnology, Changsha, China), 1 μL of each primer (10 μmol L^−1^) and 1 μL of DNA template (approximately 50 ng). The PCR amplification procedure was as follows: 94 °C for 30 s, followed by 32 cycles at 98 °C for 10 s, 58 °C for 30 s, 72 °C for 1 min and, finally, 72 °C for 2 min. The PCR products were separated by 1% agarose gel electrophoresis and purified using AxyPrep^TM^ DNA Gel Extraction Kit (Corning, Suzhou, China). Then, the purified DNA fragments were inserted into pMD19T vectors and sequenced by Sangon Biotech Company (Shanghai, China).

### 4.3. Bioinformatics Analysis

The nucleotide sequence and deduced amino acid sequence of LvMR were analyzed by using the online BLAST program (http://blast.ncbi.nlm.nih.gov/Blast.cgi (accessed on 20 September 2022)). The deduced amino acid sequence of LvMR was obtained with ORF finder (https://www.ncbi.nlm.nih.gov/orffinder/ (accessed on 20 September 2022)). The protein domain was predicted by SMART (http://smart.embl.de (accessed 20 September 2022)). The molecular weight (Mw) and isoelectric point (pI) were predicted by ExPASy ProtParam tool (https://web.expasy.org/compute_pi/ (accessed on 20 September 2022)). The potential glycosylation site was predicted by the NetNGlyc-1.0 server (https://services.healthtech.dtu.dk/service.php?NetNGlyc-1.0 (accessed on 20 September 2022)). Multiple alignment was performed with ClustalW (http://www.clustalw.org (accessed on 20 September 2022)). The phylogenetic tree based on the amino acids was constructed using MEGA 7.0 software with the neighbor-joining method.

### 4.4. Semi-Quantitative RT-PCR

The mRNA expression of LvMR in various tissues was determined by specific primers LvMR_qF and LvMR_qR using semi-quantitative RT-PCR. The cDNA product was prepared by diluting 10 times with deionized water. PCR was performed in 94 °C for 30 s, followed by 30 cycles at 98 °C for 10 s, 58 °C for 30 s, 72 °C for 30 s and, finally, 72 °C for 2 min. The 18S rRNA gene was amplified as a partial gene fragment of 147 bp using the primer pair 18S_F and 18S_R (Table 1) as an internal control. The amplification products were detected by electrophoresis on 2% agarose gel.

### 4.5. Real-Time Quantitative PCR Analysis (qRT-PCR)

The temporal expression levels of LvMR in the hepatopancreas post microorganism stimulation were detected by SYBR Green-based qRT-PCR. The cDNA product was prepared by diluting 40 times with deionized water. PCR was performed in a 10 µL reactive system, containing 2.28 µL of sterile distilled H_2_O, 3.33 µL of 2 × SYBR Premix Ex Taq (TaKaRa, Kyoto, Japan), 0.13 µL of 50 × ROX Reference Dye, 0.13 µL of each primer (10 µmol L^−1^), and 4 µL of the diluted cDNA. The PCR program was 95 °C for 30 s, followed by 40 cycles of 95 °C for 5 s and 60 °C for 35 s. For melt curve analysis, the PCR products were heated from 60 to 95 °C uniformly, and the fluorescent signal collected at every 0.1 °C rise in temperature. Each sample was taken in triplicate. The relative expression of LvMR was calculated by 2^−ΔΔCt^ method [28] using the 18S rRNA gene as internal standardization. All data were analyzed via one-way ANOVA test using SPSS 25.0, and the difference was considered significant if *p* value was less than 0.05.

### 4.6. Recombinant Expression and Protein Purification

A pair of primers LvMR_ReF and LvMR_ReR (Table 1) were designed to obtain the sequence encoding mature peptide of LvMR. The expression vector pET32a was digested by restriction enzymes *EcoR* I and *Hind* III (TaKaRa, Dalian, China). The purified PCR fragment was linked into the linearized pET32a vector using In-Fusion HD Cloning Kit (TaKaRa, Mountain View, CA, USA). The successfully sequenced recombinant expression plasmids pET32a-LvMR and pET-32a (empty vector) were transferred into *E. coli* BL21 (DE3) competent cells (Transgen, Beijing, China). The expression of rLvMR was induced by addition of IPTG to a final concentration of 0.5 mmol L^−1^ for 4 h at 37 °C. The recombinant LvMR protein were purified by TALON Metal affinity resin (Clontech, Mountain View, CA, USA) under denaturing condition. The purified proteins were refolded in gradient urea-TSB glycerol buffer according to the method by [19]. The protein solutions were concentrated with Amicon Ultra Centrifugal Filter (Millipore, Cork, Ireland). The concentration of rLvMR and rTrx were measured by BCA Protein Assay Kit (Beyotime, Shanghai, China), respectively.

### 4.7. PAMP Binding Assay

The PAMP binding assay was conducted by ELISA following previously described procedures [29] with some modification. In brief, 20 μg lipopolysaccharide (LPS, Dulai, Nanjing, China), peptidoglycan (PGN, Dulai, Nanjing, China), glucan (GLU, Yuanye, Shanghai, China) and polyinosinic acid: polycytidylic acid (poly(I:C), Yuanye, Shanghai, China) in 100 μL of carbonate-bicarbonate buffer (50 mmol L^−1^, pH 9.6) were coated to 96-well microtiter plates (NEST, Shanghai, China) at 4 °C coated overnight, respectively. After pouring out the uncoated PAMP, the plates were washed three times with PBS-T (pH 7.5) and blocked with 3% BSA in PBS for 1 h at 37 °C. Then, 100 µL diluted rLvMR was added to the wells in the presence of 0.1 mg mL^−1^ BSA and 5 mmol L^−1^ CaCl_2_ and incubated for 3 h at 18 °C. The gradient dilution of rTrx was used as a negative control. After washing three times, the plates were incubated with 100 µL of mouse anti-His tag primary antibody (Transgen, Beijing, China) at a dilution of 1:1000 in PBS with 1 mg mL^−1^ BSA for 1 h at 37 °C. The plates were washed again, and 100 µL of goat-anti-mouse Ig-HRP conjugate (Transgen, Beijing, China) at a dilution of 1:1000 in PBS with 1 mg mL^−1^ BSA was added as secondary antibody and incubated for 45 min at 37 °C. After washing the excess antibody, the chromogenic reaction was performed using EL-TMB chromogenic Kit (Sangon, Shanghai, China). Each experiment was repeated in triplicate.

### 4.8. Microorganism Agglutination and Agglutination Inhibition Assays

The experimental method of microorganism agglutination was modified according to the previous method [30]. The Gram-negative bacteria *V. parahaemolyticus*, *V. alginolyticus* and *P. aeruginosa*, Gram-positive bacteria *M. luteus* and *S. aureus*, and fungus *P. pastoris* cultured to logarithmic growth phase were collected. The microorganisms were rinsed three times with PBS and labeled with 0.1 mg mL^−1^ FITC. The excessive FITC was removed with PBS, and the bacterial concentration was adjusted to 1 × 10^8^ cfu mL^−1^ with Tris-HCl. The recombinant protein solution (0.2 mg mL^−1^) containing 10 mM CaCl_2_ was added to the microorganism solution in a 2.5:1 volume ratio, and incubated for 2 h. To characterize the Ca^2+^ dependent activity of the microorganism agglutination, 5 mmol L^−1^ EDTA (final concentration) was added to the mixture of proteins and microorganisms. The rTrx group and Tris-HCl group were used as a negative and blank control, respectively.

Ten carbohydrates were used to explore the carbohydrate binding specificity of rLvMR, including _D_-galactose (Yuanye, Shanghai, China), _D_-mannose (Yuanye, Shanghai, China), _D_-glucose (Hushi, Shanghai, China), sucrose (Hushi, Shanghai, China), LPS (Dulai, Nanjing, China), peptidoglycan (Dulai, Nanjing, China), *N*-acetylneuraminic acid (Yuanye, Shanghai, China), *N*-acetyl-_D_-mannosamine (Yuanye, Shanghai, China), *N*-acetyl-β-_D_-galactosamine (Yuanye, Shanghai, China), *N*-acetyl-_D_-glucosamine (Yuanye, Shanghai, China). The carbohydrate concentration was adjusted to 25, 50, 100 and 200 mmol L^−1^. The carbohydrate solution (20 µL) was mixed with 25 µL rLvMR and 10 mmol L^−1^ CaCl_2_ for 30 min. Then, FITC-labeled *V. parahaemolyticus* (1 × 10^8^ cfu mL^−1^) was added to the mixture. After incubation for 4 h, the mixture was detected under a fluorescence microscope. The inhibitory effect was recorded as the minimum concentration required for complete inhibition of the agglutinating activity against FITC-labeled *V. parahaemolyticus*.

### 4.9. Double Strand RNA (dsRNA) Synthesis and RNA Interference Assay

The dsRNA of LvMR were designed and evaluated by E-RNAi version 3.0 (https://www.dkfz.de/signaling/e-rnai3/ (accessed on 15 October 2022)). A 496 bp fragment of LvMR was amplified using primers LvMR_dsF1 and LvMR_dsR1 with T7 promoter based on template pMD19-T-LvMR (Table 1). The fragment was detected by 1% agarose gel electrophoresis, and purified by SteadyPure PCR DNA Purification Kit (Accurate Biology, Changsha, China). The dsRNA of LvMR was synthesized based on the purified fragment as template using Transcription Factor T7 High Yield Transcription Kit (Thermo Fisher Scientific, Waltham, MA, USA), and purified by phenol-chloroform extraction. The dsRNA concentration was measured through Nanodrop 2000 spectrophotometer (Thermo Fisher Scientific, Waltham, MA, USA). The quality of dsRNA was detected by 1% agarose gel electrophoresis. A synthetic dsRNA-EGFP was used as a control.

In RNA interference (RNAi) assay, shrimp were divided into six groups with 9 shrimp in each group. Three groups of experimental shrimp were injected with three doses of dsRNA-LvMR (0.2, 0.4 and 0.8 μg dsRNA/g shrimp weight), and three groups of control shrimp were injected with three doses of dsRNA-EGFP (0.2, 0.4 and 0.8 μg dsRNA/g shrimp weight). Each group had three biological replicates, and each replicate had three shrimp. After 48 h post dsRNA injection, hepatopancrease were collected and the interference efficiency of dsRNA was determined with primers LvMR_dsF2 and LvMR_dsR2.

### 4.10. Expression Changes of Immune Genes in the LvMR-Knockdown Shrimp

The expression of several immune genes were determined in the hepatopancrease of the LvMR-knockdown shrimp by qRT-PCR. The immune-related genes included four phagocytosis-related genes LvArf (ADP ribosylation factor 4, MK471369.1), LvRab6A (Rab GTPase, JX073679.2), LvRab (Rab GTPase, KJ742828.1) and LvRan (Ras-like nuclear protein, JX644455.1), six NF-κB pathway genes LvToll1 (DQ923424.1), LvToll2 (JN180637.1), LvToll3 (JN180638.1), LvCactus (JX014314.1), LvDorsal (FJ998202.1) and LvRelish (EF432734.1), two JAK/STAT pathway genes LvJAK (Janus kinase, KP310054.1) and LvDOME (Domeless, KC346866.1), three inflammation-related genes included LvLITAF (lipopolysaccharide-induced TNF-α factor, JN180640.1), LvIL-16 (interleukin-16-like protein, KY052164.1) and LvTRAF6 (tumor necrosis factor receptor-associated factor 6, HM581680.1), and six redox genes LvNOX (NADPH oxidase, XM_027352105.1), LvDOUX (Dual oxidase, XM_027360938.1) LvSOD, (superoxide dismutase, DQ005531.1), LvGPx (glutathione peroxidase, AY973252.2), LvGST (glutathione S-transferase, AY573381.2) and LvNOS (nitric oxide synthase, GQ429217.1).

### 4.11. Assay of Reactive Oxygen Species (ROS)

To investigate the involvement of LvMR in ROS production, hepatopancrease collected from the knockdown and control shrimp after *V. parahaemolyticus* injection were assayed for ROS levels using the ROS Assay Kit (Beyotime Biotechnology, Shanghai, China) based on the 2′,7′-dichlorodihydrofluorescein diacetate (DCFH-DA) fluorescent probe. Briefly, shrimp receiving the injection of PBS, dsEGFP or dsLvMR were injected with *V. parahaemolyticus* at a final concentration of 5 × 10^4^ cfu/tail. Each group had three biological replicates, and each replicate had three shrimp. At 6, 12 and 24 h post-injection, hepatopancreas tissues were collected from each shrimp and homogenized with chilled PBS. After centrifugation, the obtained supernatant were incubated with 5 μmol L^−1^ DCFH-DA solution for 30 min at 37 °C. Fluorescence intensity (FI) was measured on a microplate reader (Tecan, Untersbergstrasse, Austria) at excitation and emission wavelengths of 485 and 538 nm, respectively. The protein concentration of cell supernatant was determined by using the Bradford Protein Assay Kit (Beyotime Biotechnology, Shanghai, China). The relative level of ROS in the samples was expressed as FI per milligram of protein. All assays were carried out in triplicate.

### 4.12. Assay of Inducible NOS (iNOS) Activity

The iNOS activity in the hepatopancrease of the knockdown and control shrimp after *V. parahaemolyticus* injection was performed using the NOS Assay Kit (A014, Jiancheng Institute of Biotechnology, Nanjing, China). It was estimated by catalysis of the reaction between O_2_ and _L_-arginine at 530 nm. The iNOS activity was recorded as units per milligram of protein. The protein concentration was measured using a Bradford Protein Assay Kit (Beyotime Biotechnology, Shanghai, China). All assays were carried out in triplicate.

## Figures and Tables

**Table 1:** Primers used in this study.

Primers Name	Sequence (5′–3′)
LvMR_F	CGGAACTACATATAAATAGGCGTCG
LvMR_R	ATTCCCCTTTCTATTCAAGCACG
LvMR_ReF	GCTGATATCGGATCCGAATTCTGCAACCCGCCGTTCACCATG
LvMR_ReR	CTCGAGTGCGGCCGCAAGCTTGAGGAACCGCAGCTTGCAGAT
LvMR_qF	CTGCGTCAGCATCTCTTTG
LvMR_qR	GGTTTCCAGTCGGTCATTG
LvMR_dsF1	TAATACGACTCACTATAGGGTACCTTGGATTCAACGCTCC
LvMR_dsR1	TAATACGACTCACTATAGGGCACCCAGTACGAGTTCGCTT
LvMR_dsF2	GGGTCCAAATGCCTCTAC
LvMR_dsR2	ATCGCAGGAAGGGAAGGA
M13_47	CGCCAGGGTTTTCCCAGTCACGAC
RV_M	GAGCGGATAACAATTTCACACAGG
T7 promoter	TAATACGACTCACTATAGGG
T7 terminator	GCTAGTTATTGCTCAGCGGT
18S_F	TATACGCTAGTGGAGCTGGAA
18S_R	GGGGAGGTAGTGACGAAAAAT
LvRab6A_qF	CGCAGCCTTATTCCCTCATAT
LvRab6A_qR	CGCACATCATCTATCCATT
LvRab_qF	GACAGTGGTGTTGGAAAG
LvRab_qR	GCCGTCTAGTTCAATTGTTCG
LvRan_qF	CCATACAAATAGAGGACCCAT
LvRan_qR	CATTCTTGTACGTGACTCTAG
LvArf_qF	GTCTTGATGCTGCTGGTAA
LvArf_qR	CAAAGATGAGACCCTGAGTA
LvLITAF_qF	GCAGTCAACGCACATGATCT
LvITAF_qR	TTGTATTTGCCCAGGAAAGC
LvIL-16_qF	AGCAAGAGCCTCGTGTCAGAC
LvIL-16_qR	CCTCCAGAGAAAAGCCCAGT
LvTRAF6-qF	ACATCACCAATCCCAGAG
LvTRAF6-qR	GTCAGCACCGCCTTTATC
LvSOD_qF	ATTGCCGCTACGAAGAAG
LvSOD_qR	AGATGGTGTGGTTCAAGTG
LvGPx_qF	GCACCAGGAGAACACTAC
LvGPx_qR	TTCCAGGCAATGTCAGAG
LvGST_qF	AGAAAAACTACCCTGTCGG
LvGST_qR	CCTTGCTCTGCGTTATCTT
LvDUOX_qF	GACTTGGCAGCAAACCTA
LvDUOX_qR	TGCGGGAAAGGTCGTAGAT
LvNOX_qF	CCAACGATGTGCCTGATAGTG
LvNOX_qR	ATGTCGGTCTTCTGAAGGGCT
LvNOS_qF	GAGCAAGTTATTCGGCAAGGC
LvNOS_qR	TCTCTCCCAGTTTCTTGGCGT
LvToll1_qF	CTATTGTGGTGCTTTCGT
LvToll1_qR	TGGAGATGTACAGTCGTAAC
LvToll2_qF	CATGCCTGCAGGACTGTTTA
LvToll2_qR	GGCCTGAGGGTAAGGTCTTC
LvToll3_qF	GTGAATCTGACCCGAGTTGA
LvToll3_qR	TGCTGCCTTCGGTGTTCTA
LvCactus_qF	GCCTGTCTTACGCCCCT
LvCactus_qR	CCGTCCGACCACTCTTG
LvRelish_qF	CATGCAAGACTTCGCAA
LvRelish_qR	CTGGTAATGTAACAGGACG
LvDorsal_qF	TGGGGAAGGAAGGATGC
LvDorsal_qR	CGTAACTTGAGGGCATCTTC
LvJAK_qF	CCTTAATTCGAGCGCAATGGG
LvJAK_qR	CTAGCGACAGAGGGTTTAGCG
LvDOME_qF	CTCAGGCTATGTTTCTCAGGATTCA
LvDOME_qR	CACGGCAGTTCCTTTATGGTCT

## Data Availability

The data presented in this study are available in the article.
